# A multilevel perspective of developmental feedback and employee creativity

**DOI:** 10.3389/fpsyg.2026.1765705

**Published:** 2026-03-05

**Authors:** Xiaolu Li, Gang Liu, Lin Sun

**Affiliations:** 1 School of Business, Nanjing University, Nanjing, China; 2 School of Business Administration, Zhongnan University of Economics and Law, Wuhan, China; 3 School of History, Culture and Tourism, Guangxi Normal University, Guilin, China

**Keywords:** developmental feedback, employee creativity, multilevel perspective, problem identification, team reflexivity

## Abstract

Drawing from the broader feedback literature and the creativity literature, we offer a multilevel perspective to examine how receiving developmental feedback could positively influence one’s creativity. We resolve a key puzzle in the feedback–creativity literature—why feedback sometimes predicts creativity and sometimes does not—by theorizing developmental feedback as a multilevel system rather than a single-source input. Integrating supervisor and team developmental feedback shows that creativity emerges when developmental cues align across levels to facilitate early-stage problem identification, thereby advancing creativity theory from outcome explanations to process-based accounts. We identified both individual-level and team-level mediating mechanisms linking developmental feedback to creativity. We collected data from a large telecommunication company in China. Hierarchical linear modeling results based on 642 employees nested in 103 teams functioning in four areas supported our hypotheses. Both supervisor developmental feedback (SDF) and team developmental feedback (TDF) were positively related to employee creativity. Employees’ problem identification mediated the relationship between SDF and creativity, while team reflexivity mediated the relationship between SDF and problem identification as well as creativity. Our findings suggest that developmental feedback that is informational, motivational, and future-oriented in nature from one’s supervisor and the team contributes to the generation of both novel and useful ideas. We also discuss implications for research and address the limitations of this research.

## Introduction

Employee creativity, defined as the generation of novel and useful ideas (Amabile, 1983, 1988), has been one of the most extensively examined topics in organizational behavior over the past two decades (Carnevale et al., 2021; George and Zhou, 2002; Jia et al., 2024; Oldham and Cummings, 1996; Tierney et al., 1999; Woodman et al., 1993; Zhou and George, 2001). As an important job design factor, feedback has been theorized as a critical driver of employee creativity. However, prior research has yielded mixed findings regarding the feedback–creativity relationship. For example, Kim and Kim (2020) found that negative feedback can exerted both positive and negative effects on employee creativity, whereas Zhou (2003) observed no significant relationship. These studies largely build on the logic that feedback may increase or decrease employees’ intrinsic motivation (George and Zhou, 2002; Shalley et al., 2004; Zhang and Bartol, 2010) or self-efficacy (Tierney and Farmer, 2002), thereby fostering employee creativity.

Despite prior studies have presented the importance of feedback for employee creativity, they yield inconsistent results largely because they have not directly tested the role of feedback in the creative process. Drawing on the componential model of creativity process (Amabile, 1983, 1996), creativity comprises a sequence of stages, including problem identification, information searching, idea generation, and idea evaluation. As the initial stage, problem identification captures employees’ capacity to accurately identify work-related problems and is essential for creativity. Accordingly, directly theorizing and testing problem identification as a mediating mechanism linking feedback to employee creativity is both necessary and timely. In addition, prior research has largely confined its attention to the relationship between feedback and creativity at the employee level, failing to consider the joint influence of team- and employee-level feedback on employee creativity. Such a narrow focus constrains a more complete understanding of how feedback operates across levels to shape creativity.

Building on this logic, the present study adopts a multilevel perspective to investigate how developmental feedback from supervisors and from team members jointly influences individual creativity. In particular, we focus on developmental feedback because it embodies the informational and future-oriented features of work contexts that facilitate creativity by helping employees understand their tasks and engage in creative processes (Amabile, 1996; Oldham and Cummings, 1996). Unlike evaluative feedback that focuses on past performance discrepancies, developmental feedback communicates specific information about how employees can improve and grow, while simultaneously conveying confidence in the employees’ potential, which fosters future-oriented behavior (Brett and Atwater, 2001; Shrauger, 1975; Zhou, 2003). These characteristics make developmental feedback especially well suited to influence problem identification (Li et al., 2011; Zhou, 2003). Additionally, the informational and motivational value of developmental feedback cannot be fully understood when it is examined solely at one level of analysis. Creativity frequently arises from the interaction between individual characteristics and collective influences (Amabile, 1996). As such, employees may need both developmental guidance from supervisors (supervisor developmental feedback, SDF) and a corresponding form of developmental communication within their team (team developmental feedback, TDF) to effectively convert feedback into creative processes. Thus, we propose that developmental feedback exerts its effects not only directly but also through specific individual-level and cross-level mediating mechanisms. By examining both supervisor and team sources of developmental feedback, and by theorizing about the pathways through which this feedback translates into creative outcomes, we contribute to a more nuanced understanding of how organizations can cultivate the informational and motivational conditions necessary for creativity to flourish.

**Figure 1:**
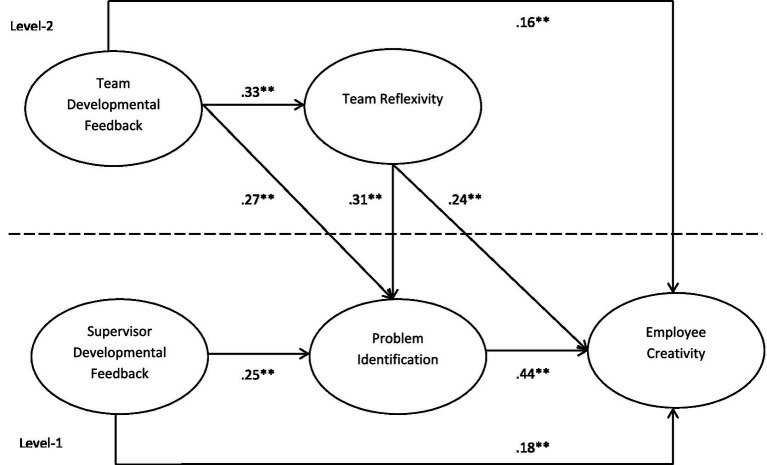
The hypothesized multilevel model of developmental feedback and creativity. *p < 0.01,**p < 0.05, ***p < 0.001.

This research makes several important contributions to the literature on feedback and creativity. First, we directly theorize and test problem identification as a core mechanism linking developmental feedback to employee creativity, allowing for a more precise capture of how and why feedback shapes employee creativity. Second, we advance prior work (Kim and Kim, 2020; Van Dijk and Kluger, 2011; Zhou, 2003; Zhang et al., 2019) by adopting a multilevel framework to examine the effects of developmental feedback on creativity from both employee and team levels. Third, we extend the feedback and creativity literature by demonstrating how team-level developmental feedback influences employees’ problem identification and, in turn, employee creativity, thereby introducing a cross-level mechanism that has received limited attention in prior studies. Figure 1 presents our full theoretical model.

## Theory and hypotheses

Consistent with prior research, in the present study we define employee creativity as the generation of novel and useful ideas concerning products, service, job procedures, and work processes (Amabile, 1983, 1988, 1996; Oldham and Cummings, 1996; Shalley, 1991). Previous research has made initial efforts to investigate the relationship between supervisor developmental feedback and employee creativity. For instance, Zhou (2003) found that the joint (i.e., interacting) condition of supervisor developmental feedback and the presence of creative coworkers contributed to the production of creative outcomes such that when creative coworkers were present, more supervisor developmental feedback led to higher level of employee creativity. Yet, Zhou (2003) did not find a direct positive relationship between supervisor developmental feedback and employee creativity, nor did the study outline any mechanism linking these two constructs. However, other studies have found that the effect of positive peer feedback on enhancing subsequent idea quality strengthens as ideators gain experience (Chan et al., 2021), though they did not examine the underlying mechanisms linking the two constructs. Meanwhile, some scholars argue that negative feedback inhibits creativity (Ilies and Judge, 2005; Van Dijk and Kluger, 2011; Zhou, 1998), while recent research suggests that even negative feedback may have constructive effects (Kim and Kim, 2020).

### Supervisor developmental feedback and employee creativity

#### An overview of supervisor developmental feedback

SDF refers to supervisors’ provision of helpful and valuable information intended to help employees “learn, develop, and make improvements on the job” (Zhou, 2003, p. 415). Extending beyond standard performance evaluations, SDF emphasizes guidance on domain-relevant skill building, on-the-job learning, goal setting, and workplace socialization (Atwater and Brett, 2005; Li et al., 2011; Zhou, 2003). Whereas traditional performance feedback focuses primarily on assessing how well employees have met job requirements, SDF elevates employees’ awareness of their work content and environment by offering future-oriented suggestions that support learning and development (Li et al., 2011; Zhang et al., 2024). Through such forward-looking input and mentoring, SDF strengthens employees’ persistence and willingness to explore (Zhou, 2003), enhances their intrinsic motivation toward their work (Li et al., 2011), and facilitates their better understanding about the meaning of their work (Zhang et al., 2024). In offering developmental feedback, supervisors not only communicate information about employees’ past performance but also expand the information stream by providing explicit, forward-looking suggestions for improvement and change (Atwater and Brett, 2005; Li et al., 2011).

#### Supervisor developmental feedback and employee creativity

This study proposes that the informational, motivational, and future-oriented characteristics of supervisor developmental feedback (SDF) are positively correlated with employee creativity. First, the enriched flow of information created through SDF exposes employees to diverse and useful insights about task characteristics, problem-solving alternatives, and the uniqueness of their task environment (Zhou, 2003). When delivering developmental feedback, supervisors often pose open-ended questions that prompt employees to reassess their task approaches, reinterpret past behaviors, and plan improved strategies for future performance (Atwater and Brett, 2005). Such descriptive—rather than evaluative—communication encourages idea generation and the accumulation of implicit knowledge that supports creative thinking (Lindgren and Lindgren, 1965; Simon, 1966). This richer informational foundation helps employees organize and, when necessary, restructure cognitive pathways to generate novel and useful ideas (Simon, 1966). In this sense, the process through which SDF influences employees could also be considered a positive self-reflection process, one that can potentially enable employees to more comprehensively understand the various aspects and meanings of their work and therefore see the true sense of purpose behind what they do (Zhang et al., 2024), which should contribute to one’s sustained commitment to identify novel ways to improve their work practices.

Second, SDF may enhance employees’ intrinsic motivation, a critical driver of creativity. A defining feature of SDF is its focus on learning and improvement, communicated through positive, constructive messages (Atwater and Brett, 2005; Li et al., 2011; Zheng et al., 2015). Because individuals respond more favorably to well-reasoned, constructive input (Shrauger, 1975), employees are likely to react positively to the specific, improvement-oriented guidance provided by supervisors. This positive feedback loop can heighten employees’ interest in the task and their willingness to explore (Brett and Atwater, 2001; Russell, 1980), thereby supporting the emergence and deepening of intrinsic motivation (Zheng et al., 2015). Furthermore, the future-oriented emphasis of SDF may promote mastery-oriented learning goals, which represent proactive efforts that facilitate creative performance (Dweck, 1986; Dweck and Leggett, 1988; Elliot and Dweck, 1988). With the above said, we propose the following hypothesis:

*Hypothesis 1*: Supervisor developmental feedback is positively related to employee creativity.

#### The mediating role of problem identification

Focusing on learning and improving, SDF enables employees to more clearly identify the nature of the tasks, the characteristics of the task environment, the future growth curve by on-the-job learning through problem-solving, and the potential room for idea exploration. Its developmental nature helps employees to recollect and reformulate information about their performed jobs (Atwater and Brett, 2005), creating a deep-thinking pattern in which employees are intrinsically motivated to understand what the nature of job problems are, why such nature matters to the achievement of task goals, what the possible resources and solutions are available, and whether there are better ways to resolve the job problems. Moreover, such intrinsic motivation is also suggested to primarily navigate individuals’ control of attention towards the object of their focus (Anderson et al., 1976; Amabile, 1988; Simon, 1966). That is, intrinsically motivated individuals are more likely to persist on identifying job problems and are more willing to be dedicated to the problem solving (Zhang and Bartol, 2010). In addition, supervisor developmental feedback may also provide employees with diversified perspective towards understanding the job tasks and the task environment (Atwater and Brett, 2005; Zheng et al., 2015; Zhou, 2003), thus enhancing their problem identification behaviors such as speaking up with improvement-oriented suggestions and ideas that could fuel up creative performance (Sibunruang and Kawai, 2024). With the above said, we propose that by offering developmental feedback, supervisors enable employees to engage more in problem identification at the workplace.

*Hypothesis 2*: Supervisor developmental feedback is positively related to employees’ problem identification.

Although much previous research has considered new idea generation as a critical element of the concept of creativity, it should be noted that the classic componential framework of creativity also highlights the “*usefulness*” of ideas as a key to justify new ideas to be representative of creative outcomes (Amabile, 1988). To determine the “*usefulness*” of ideas not only requires involvement in work processes, but also demands a deep understanding on the nature and the mechanism of the job tasks (Mumford, 2000; Shalley et al., 2004). Without a thorough understanding on the job tasks and the task environment, it is less likely for employees to produce novel and useful ideas that can be later applied to their jobs.

Zhang and Bartol (2010) conceptualized problem identification as a major dimension of creative process engagement since it captures the uniqueness of creativity-relevant cognitive processes aiming at understanding the nature and the mechanism of the tasks. First, problem identification reflects employees’ motivation to engage in the job by decomposing the requirement and the fulfillment of job tasks, re-analyzing the performed task procedures, and re-evaluating the task environment (Simon, 1966; Van de Ven and Delbecq, 1971). Second, problem identification incorporates multiple and flexible cognitive path ways through which employees direct their attention to diversified aspects of the task (Amabile, 1996). It thus may enrich employees’ understanding towards the characteristics of the job tasks and develop novel perspectives. Third, problem identification is considered a form of conscious mental activity that carries out committed interpretation of the job tasks and the task environment (Simon, 1966). The repeated scanning of the job tasks may enrich the assessment of reformulated information that is relevant to produce novel ideas (Zhang and Bartol, 2010). Therefore, we propose that supervisor developmental feedback (SDF) is associated with employee creativity through employees’ deep reevaluation of past job performance, repeated interpretation of tasks and the task environment, and its connection to the development of a more flexible cognitive pattern, which in turn facilitates the generation of creative outcomes. Based on this, we hypothesize the following:

*Hypothesis 3a*: Problem identification is positively related to employee creativity.

*Hypothesis 3b*: Problem identification partially mediates the positive relationship between supervisor developmental feedback and employee creativity.

### Team developmental feedback and employee creativity

#### The foundation of team developmental feedback

The existing literature on developmental feedback primarily rests on two sources: supervisor developmental feedback and coworker developmental feedback (CDF, Li et al., 2011; Zhou, 2003). Both of these two concepts are conceptualized and operationalized at the individual level of analysis (Li et al., 2011). Previous research suggests that developmental feedback from a rich body of coworkers in the workplace contributes to the accurate evaluation of feedback information and thus enable employees to develop ideas that are more likely to be practically useful (Brett and Atwater, 2001). Yet, there is a lack of understanding in the nature and the property of developmental feedback at the team level. In the present study, we aim at offering a multilevel perspective linking developmental feedback at both individual (i.e., supervisor as the source) and team level with employee creativity. In the later section, we first outline the characteristics of TDF and then address the mechanism through which TDF might impact individual-level creative outcomes.

Consistent with prior research in defining SDF (Zhou, 2003), we define TDF as *a form of feedback through which each member of the team provide other team members with helpful or valuable information that enables other team members to learn, develop, and make improvements on the job*. Team developmental feedback is a team-level construct that captures a shared team process—specifically, a recurring and collective pattern of developmental feedback exchange among team members. It reflects the team’s overall feedback practice (“how feedback is typically exchanged within the team”) and constitutes a common interaction pattern and informational environment shared by the team. In contrast, coworker (individual) developmental feedback is an individual-level construct, which describes the extent to which a particular employee directly receives developmental feedback from colleagues. As it is based on personal experience of receiving feedback, it may vary significantly among members within the same team. In line with this definition, we propose three unique characteristics of this concept. First, TDF is informational and future-oriented in nature. When team members offer developmental feedback to each other, this team is essentially engaging in a progressive process of information enrichment. That is, each member of the team provides other members with behaviorally relevant information that may contribute to the development of domain-relevant skills and is associated with the improvement of job performance in the future. Such information enrichment at the team level is different from that at the individual level (i.e., CDF) in that the former offers a context for diversified information exchange among multiple agents in the focal team, while the later only focuses on developmental feedback directed from coworkers to self.

Second, TDF functions as a motivational context for the facilitation of team members’ intrinsic motivation towards job tasks. Aligned with the intrinsic motivation perspective (Deci and Ryan, 1985), TDF leverages the intrinsic motivation of team members by creating a context where team members primarily focus on learning and improving. The traditional feedback-seeking perspective emphasizes that individuals tend to seek feedback to balance the discrepancies between past performance and the set goals (Ashford, 1986; Ashford and Cummings, 1985; Kluger and DeNisi, 1996). In contrast, we suggest that TDF focuses on fostering employees’ orientation toward learning and improvement with a consciously self-directed process of progressive change in the future. Such progressive change rests on repeated interpretation of information cues, continuous reorganization of existing knowledge, content-based imitation of better-performing models, and self-developed cognitive and behavioral patterns. These progressive changes are made possible by team members’ use of future-oriented descriptive feedback aimed at helping others to improve rather than past-oriented judgmental feedback focusing merely on performance evaluation.

Finally, we propose that in the team context TDF incorporates a repeated socialization process through which information exchange with an orientation of mutual-learning and mutual-improvement enhances team members’ willingness to engage in collaborative team efforts. With this said, TDF is different from the traditional content-based feedback (e.g., social or normative feedback) in that the content-based performance feedback focuses on specific task information (Ashford et al., 2003), while TDF contains multiple information cues beyond only job tasks but rather from the positive social interactions among team members. Thus, TDF offers a platform for within-team exchange of both in-role and extra-role expectations, relational networking, and skill-relevant mutual-learning. Teams high in TDF should hold a shared belief about the positive impact of collaborative team efforts so that team members are committed to engaging in such efforts as to produce better future outcomes.

#### Team developmental feedback, problem identification, and creativity

The characteristics of TDF as stated above indicate that TDF might contribute to the generation of new and useful ideas (i.e., employee creativity) through employees’ development of intrinsic motivation on the task and the interactions of their diversified cognitive patterns. That is, TDF is likely to be perceived by team members as a motivational context which encourages them to engage in the job with by learning from the behavioral and cognitive patterns of the role models in the team, improving future performance based on within-team benchmarking, and interacting with each other through both task and social communication. These important attributes of a developmental team context are likely to be associated with employees’ deep thinking towards understanding the nature of performed tasks and progressive exploration of potential avenues for future improvement.

TDF may both directly facilitate the emergence of creativity and influence so by triggering employees’ problem identification behaviors. First, by nature TDF not only helps to develop employees’ intrinsic motivation towards performing tasks but also leads to continuous interactions among team members with an orientation of information exchange and mutual-learning. The information enrichment and mutual-learning orientation altogether might encourage employees in raising ideas that are not only novel based on the enriched informational environment, but also useful based on their deeper understanding on the task. And the motivational aspect of TDF is likely to enable employees to persist on carrying out new idea generation process. Second, the social interactions enriched in the TDF context are likely to encourage team members to benchmark their own job behaviors and performance with those of role models in the team, while benchmarking in a collaborative context (such as TDF) is suggested to be associated with employees’ proactive engagement in identifying problems and discovering new pathways to perform better in the future (Cox et al., 1997). Moreover, employees’ comparisons on diversified perspectives, along with the motivational influence of TDF, may also enable them to rethink the traditional way of performing jobs, identify the existing problems that hinder superior performance, and search for new aspects that may help them to improve in the future. With the above said, we propose that TDF positively relates to creativity through employees’ problem identification while also imposing direct influence to produce creative outcomes.

*Hypothesis 4a*: Team developmental feedback is positively related to employee creativity.

*Hypothesis 4b*: Problem identification partially mediates the positive relationship between team developmental feedback and employee creativity.

#### The mediating role of team reflexivity

In the present study, as we investigate creativity in a developmental feedback context which is informational, motivational, and future-oriented in nature, we expect to identify collective behaviors at the team level to leverage the characteristics of such developmental context in producing individual-level creative outcomes that are not only novel but also practically useful. With this said, team reflexivity (West, 1996), a construct incorporating both task and process reflexivity within a team, may well serve our research purposes. Team reflexivity refers to the extent to which teams reflect upon and modify their functioning (West, 1996). By doing so, team members adapt team’s objectives, strategies, and processes to face future challenges and uncertainties (Tjosvold et al., 2004; West, 2000). Its concept rests on the notion that the environment of a team is ever changing and thus requires constant reflection and contemplation to assess the environment and deliver appropriate actions (Carter and West, 1998; West, 1996).

By definition, team reflexivity is more likely to attend to a developmental feedback context because both of these two constructs emphasize future-oriented actions and information exchange among team members. It is also associated with individual behaviors aimed at identifying and resolving problems in the focal context (Carter and West, 1998; West, 2000). As described earlier, TDF is characterized by within-team information enrichment process through which diversified information that is behaviorally relevant to performing jobs is exchanged among team members. Such information exchange not only helps team members to reformulate information pertaining to their own performance, but also enables them to recognize the discrepancies between individual job behaviors and behaviors that are expected for team task collaboration (Dominick et al., 1997). Therefore, team members are more likely to raise collective awareness of the need for improvement in the future and reflect on current objectives and approaches. On the other hand, the future-oriented nature of TDF encourages constant development of new understandings towards job tasks and the task environment. Given that TDF offers a cooperative context where repeated socialization process enhances mutual-learning and collaborative task engagement, team members are likely to rely on the relational networking from the socialization process to collaboratively and constantly comprehending team tasks and the task environment (Li et al., 2011). With the above said, we propose the following hypotheses:

*Hypothesis 5a*: Team developmental feedback is positively related to team reflexivity.

*Hypothesis 5b*: Team reflexivity partially mediates the positive relationship between team developmental feedback and problem identification.

Previous research has identified team reflexivity as an importance source for team innovation (Tjosvold et al., 2004; West, 2000). It is suggested that reflexive teams tend to constantly respond to emerging conditions that are uncertain and, sometimes, challenging in nature (Carter and West, 1998; De Dreu, 2007; Hirst and Mann, 2004). By closely monitoring the environment and modifying objectives and task approaches to reflect on the emerging conditions, reflexive teams are likely to produce innovative outcomes (Hoegl and Parboteeah, 2006; West, 2000).

In the present study, we incorporate team reflexivity in a developmental feedback context which shares similar contextual characteristics (e.g., information exchange, future-oriented). We extend the role of team reflexivity to cross-level processes and suggest that team reflexivity may also relate to individual-level creative outcomes, thereby leveraging the characteristics of team developmental feedback to foster such outcomes. First, the developmental feedback given by each member of the team promote job-related discussions in which the raise of open-ended questions and descriptive evaluations of existing problems might raise employees’ self-awareness of potential new pathways to perform the job (Hoegl and Parboteeah, 2006). Second, team reflexivity emphasizes the contemplation on tasks and processes especially when a team runs into situations with uncertainties (Carter and West, 1998; West, 1996, 2000). Such contemplation is suggested to relate to employees’ deep thinking regarding their own involvement in performed tasks (West, 1996). Given the rich informational exchange that they are supposed to receive from other team members in a developmental feedback context, employees are thus likely to develop new and practically useful ideas to respond to specific task procedure or job content. Third, team reflexivity incorporates exploratory learning and progressive questioning as collective efforts to scan the existing job tasks and the task environment and to respond to emerging conditions with uncertainties (Tjosvold et al., 2004; West, 1996, 2000). Therefore, in reflexive teams employees are likely to rely on an explorative approach to search for relevant information, decode information cures, and develop novel perspectives that align with the needs of their jobs. With the above said, we propose that team reflexivity leads to individual creativity and mediates the relationship between TDF and creativity.

*Hypothesis 6a*: Team reflexivity is positively related to employee creativity.

*Hypothesis 6b*: Team reflexivity partially mediates the positive relationship between team developmental feedback and employee creativity.

## Methods

### Sample and procedure

We invited employees working at a large Chinese telecommunication company to participate in the present study. The invited employees worked in teams that function in four areas, including research and development (R&D), business process consulting (BPC), sales, and key customer service (KCS). Members of these teams all performed tasks involving the development of new ideas and approaches to attract and retain external customers or serve the interest of internal customers. There was one supervisor (team leader) assigned to manage each team. Team members frequently engaged in collaborative tasks requiring collective efforts to achieve the team goals. Regular team meetings were set up at both the beginning and the end of each week so that team members could preview the new expected objectives, review the task progress, and discuss the potential for performance improvement. Moreover, at the beginning of each month, team leaders also provided each team member a formal work review, in which team leaders identified key performance index and offered advice for improvement based on individual performance of the previous month. These work settings indicated that both the within-team developmental feedback and supervisor developmental feedback might produce relevant conditions to encourage employee creativity.

We invited all 126 supervisors in charge of the four types of teams as identified above as well as 891 team members working in those 126 teams. We briefed the participants about the purpose of this study and explained the procedures for completing on-line surveys. We emphasized that their participation should be completely voluntary and that the Company would not have the access to any identifiable information. To better protect the confidentiality of participants, we also assigned random identification numbers to each invited participant so that we could later on match up each number with a specific survey participant. Of the 891 invited employee participants, 687 completed self-report on-line surveys, yielding an initial response rate of 77.1%. Of the 126 supervisors, 109 completed on-line surveys providing ratings on employees’ creativity, yielding an initial response rate of 86.5%. Given that one of our research purposes was to explore how developmental feedback that team members offered to each other might impact individual creativity, we expected for a relatively high team response rate to really capture the characteristic of a team context enriched with or in lack of developmental feedback given by each team member. Previous team research suggested that a team response rate of <60% might result in measurement errors to produce type II errors for the aggregation of data to team level (Baruch, 1999; Roth and BeVier, 1998; Timmerman, 2005). Therefore, in the present study, we decided to use 70 percent as the cut-off team response rate to justify the representativeness of team-level responses. After omitting teams lacking adequate observations and matching up supervisor responses with employee responses, we finalized with a sample of 642 employees nested in 103 teams, including 38 R&D teams, 29 BPC teams, 24 sales teams, and 12 KCS teams. Team size ranged from 4 to 9 members, with an average of 6.2 members. Among the 642 employee respondents, 182 were female (28.3%). The average age and organizational tenure for all employee respondents in our sample were 30.4 and 5.4 years, respectively. All employee respondents were reported to have at least a bachelor degree, while 168 (26.2%) of them held a master degree or above. The average age of 103 supervisors was 34.8 years. Among them, 77 (74.8%) were male. The average organizational tenure of supervisors was 8.3 years.

### Measures

We followed a translation/back-translation procedure (Brislin, 1986). Specifically, we first translated all English items into Chinese, and then independently back-translated them into English. The translation and back-translation were conducted by two doctoral students and one associate professors with expertise in organizational behavior and bilingual proficiency. The translators compared the back-translated version with the original items, discussed discrepancies, and refined wording until they reached consensus on semantic equivalence, clarity, and contextual appropriateness for Chinese respondents. We used 7-point Likert scale ranging from 1 (strongly disagree) to 7 (strongly agree) to measure all variables in the present study.

***Supervisor developmental feedback*:** SDF was assessed with a 3-item scale developed by Zhou (2003). A sample items is “While giving me feedback, my supervisor focuses on helping me to learn and improve”. The Cronbach’s alpha was 0.89.

***Team developmental feedback*:** TDF was assessed with a 4-item scale developed from Zhou and George’s (2001) coworker developmental feedback scale. Items included: “The members of this team provide each other with valuable information about how to work better to improve performance”, “The members of this team discuss open-ended questions that encourage each other to reformulate information relevant in performing job tasks”, “While giving each other feedback, the members of this team focus on helping each other to learn and improve”, and “While giving each other feedback, the members of this team provide opinions primarily by using descriptive rather than judgmental words”. The Cronbach’s alpha was 0.91. To check the validity of this scale, we further conducted a confirmatory factor analysis (CFA). All items loaded well on the scale with factor loadings ranging from 0.82 to 0.87. The CFA results revealed good fit of the TDF scale based on the recommended fit indices (Hu and Bentler, 1999): *χ*^2^ = 5.76, df = 2, *p <* 0.01, comparative fit index (CFI) = 1.00, Tucker-Lewis index (TLI) = 0.99, root mean square error of approximation (RMSEA) = 0.03, standardized root mean square residual (SRMR) = 0.01.

***Problem identification*:** We measured employees’ problem identification by using the 3-item problem identification dimension of Zhang and Bartol’s (2010) creative process engagement scale (CPE, consisting of three dimensions: problem identification, information searching and encoding, and idea generation). Respondents were asked to answer the following question: “In your job, to what extent do you engage in the following actions when seeking to accomplish an assignment or solve a problem?” A sample item was “I decompose a difficult problem/assignment into parts to obtain greater understanding”. The Cronbach’s alpha was 0.87.

***Team reflexivity*:** Team reflexivity was assessed with a 7-item scale developed by Hirst and Mann (2004). This scale consisted of two dimensions: task reflexivity and process reflexivity. A sample item of task reflexivity was “This team steps back from daily routines to consider whether the methods used are the best available”. A sample item of process reflexivity was “The members of this team regularly discuss whether the team is working effectively together”. The Cronbach’s alpha was 0.91.

***Employee creativity*:** Creativity was assessed with a 9-item scale developed by Tierney et al. (1999). A sample item was “This employee generated novel but operable work-related ideas”. The Cronbach’s alpha was 0.92.

***Control*** var***iables*:** Consulting with previous studies in creativity, we controlled for employees’ age, gender, organizational tenure, and their educational background at the individual level. At the team level, we also controlled for the type of teams given that in this study we surveyed four types of teams.

### Aggregation tests

Scores of TDF and team reflexivity were both aggregated from individual ratings to the team level. In support of aggregation, the median r_wg(j)_ across the team was 0.98 for team developmental feedback and 0.97 for team reflexivity, indicating that members of the teams in the present study shared common perceptions regarding the developmental feedback and team reflexivity in their particular teams (James et al., 1984). We obtained additional support for aggregating these two team measures by checking the intraclass correlations (ICC). We used HLM 6.08 (Raudenbush and Bryk, 2002) to run null models in which we included TDF and ream reflexivity as dependent variables, respectively, with no predictor added. The ICC1 and ICC2 values for TDF and team reflexivity were [0.32, 75] and [0.38, 0.79], respectively. This indicated that 32% of the variance in TDF and 38% of the variance in team reflexivity were explained by between-team effects (level-2). In addition, we also performed one-way analysis of variance (ANOVA) which showed that there were significant differences in team-level means of TDF, *F*(102, 539) = 4.07, *p <* 0.001, and team reflexivity, F(102, 539) = 4.85, *p <* 0.001. Taken together, the above line of evidence supported the appropriate aggregation of TDF and team reflexivity (Bliese, 2000).

### Analytical strategy

We employed four steps to analyze the data. First, before testing our hypotheses, we conducted a series of CFAs to test the validity of our studied constructs and to confirm whether these constructs altogether conformed to the established theory. Second, given that the data collected attended to a hierarchical structure in which employee respondents were nested in teams, we used hierarchical linear modeling (Raudenbush and Bryk, 2002) to estimate the hypothesized multilevel relationships. Specifically, we used HLM 6.08 to partition and estimate the within-team effects (level-1) and the separate effects of team-level predictors (level-2) on the intercepts and slopes at the individual level. Such multilevel modeling technique provides more accurate interpretations of the model in that it captures the correlations among the level-1 observations through the estimation of random effects across level-2 units/teams (Bauer et al., 2006; Raudenbush and Bryk, 2002). In the present study, we established a theoretical model integrating 1–1-1, 2–1-1, and 2–2-1 mediation models. We thus followed recommendations from Zhang et al. (2009) to integrate the testing of these lower-level and upper-level mediation models. We also consulted with Bauer et al. (2006) to calculate the random indirect and total effects of lower-level and upper-level mediation models. Third, of the two ways to calculate mediation effects (*product-of-coefficient* and *difference-in-coefficient*), we chose the *difference-in-coefficient* method (independent variables affecting dependent variables), because it enabled us to estimate the total mediation effects of a specific mediator in multilevel models while partitioning level-1 and level-2 mediation effects (Krull and MacKinnon, 1999; Zhang et al., 2009). Last, we used the Monta Carlo method recommended by Zhang et al. (2009) and Preacher et al. (2010) to estimate the confidence intervals (CI) for the hypothesized multilevel mediated relationships as to determine the significance of mediation effects. Such method was also used in previous studies investigating cross-level mediating relationships (e.g., Zhou et al., 2012).

A critical concern that has been raised in conducting multilevel mediation analysis rests on the choice of centering method for the specific mediation models (Hofmann and Gavin, 1998; Preacher et al., 2010; Zhang et al., 2009). Using inappropriate centering technique might produce confounded estimates of the mediation effects at different levels (Hofmann and Gavin, 1998; Zhang et al., 2009). In the present study, we primarily used group-mean centering method because it enabled us to partition team-level (level-2) effects of the predictors from their within-team (level-1) effects on the criterion variables in 1–1-1 models and more accurately interpret the team-level (level-2) mediation effects in 2–1-1 models (Zhang et al., 2009). The grand-mean centering method as normally used on level-1 variables, in contrast, might produce confounded estimates of level-1 and level-2 mediation effects especially in 1–1-1 and 2–1-1 mediation models (Zhang et al., 2009). We therefore addressed three different sets of model testing (i.e., 1-1-1, 2-1-1, 2-2-1) as shown in Table 1 with employee creativity as the dependent variable.

**Table 1:** HLM results: the effects of developmental feedback on employee creativity.

Variables	Employee creativity					
	1-1-1 model		2-1-1 model		2-2-1 model	
	Model 4	Model 5	Model 6	Model 7	Model 8	Model 9
Intercept	4.42**	4.42**	4.44**	4.44**	4.44**	4.44**
Level-1 control						
Age, γ_10_	0.01	0.01	0.01	0.01	0.01	0.01
Gender, γ_20_	−0.03	−0.04	0.03	0.00	−0.00	−0.01
OrgTen, γ_30_	0.03	0.03	0.03	0.04	0.03	0.03
Edu, γ_40_	0.04	0.05	0.04	0.05	0.07	0.09*
Level-1 predictor						
SDF^Ϯ^, γ_50(1)_	0.58**					
SDF^Ϯ^, γ_50(2)_		0.47**				
SDF, γ_50(3)_			0.25**	0.21**	0.19**	0.18**
PIden^Ϯ^, γ_60(1)_		0.44**		0.42**		
PIden, γ_60(2)_					0.37**	0.35**
Level-2 control						
Type of team, γ_01_	0.05	0.06	0.04	0.05	0.02	0.01
Level-2 predictor						
TDF^Ϯ^, γ_02_	0.25**	0.23**	0.28**	0.16**	0.30**	0.21**
Team Reflexivity^Ϯ^, γ_03_	0.36**	0.31**	0.31**	0.30**		0.24**
SDFmean, γ_04_	0.16**	0.12**				
PIdenmean, γ_05_		0.23**		0.09**		
Pseudo R^2^	0.21	0.34	0.17	0.28	0.19	0.26

Level 1 N = 642 (level 2 N = 103). OrgTen: Organizational tenure; Edu: Educational level; SDF: Supervisor developmental feedback; PIden: Problem identification; TDF: Team developmental feedback; SDFmean: Group mean of supervisor developmental feedback; PIden mean: Group mean of problem identification. Pseudo R^2^ was calculated based on the proportional reduction of Level 1 and Level 2 error variance due to predictors (Snijders and Bosker, 1999). ^Ϯ^The variables were group-mean centered. *p < 0.05, **p < 0.01.

## Results

Table 2 provides the descriptive statistics, correlations and Cronbach alpha for the studied variables. In general, the strength and the direction of reported correlations among variables were as expected. The bivariate associations shown in the correlation matrix rendered initial support for main effects among individual-level variables. Specifically, SDF was positively related to employees’ problem identification (r = 0.39, *p <* 0.01) and creativity (r = 0.32, *p <* 0.01), while problem identification was also positively related to creativity (r = 0.45, *p <* 0.01). The Cronbach’s alpha values shown in Table 2 ranged from 0.87 to 0.92, indicating good internal consistency of the items for each measure.

**Table 2:** Descriptive statistics and correlations of studied variables.

Variables	Mean	S.D.	1	2	3	4	5	6	7	8	9	10
1. Age	30.38	3.50	–									
2. Gender	1.72	0.45	0.05	–								
3. OrgTen	5.36	2.18	0.51**	−0.02	–							
4. Education	2.31	0.55	−0.07	−0.07	0.00	–						
5. Type	2.10	1.03	0.01	−0.02	0.02	−0.04	–					
6. SDF	4.50	1.25	−0.07	0.01	−0.07	−0.01	0.03	(0.89)				
7. PIden	4.44	1.00	0.07	0.04	0.01	−0.06	−0.02	0.39**	(0.87)			
8. TDF	4.60	1.37	0.04	0.01	−0.03	0.11**	−0.02	0.24**	0.38**	(0.91)		
9. TR	4.34	1.16	0.17**	0.04	0.04	0.05	0.02	0.27**	0.41**	0.49**	(0.91)	
10. Creat	4.42	1.28	0.10*	0.00	0.05	0.08*	−0.04	0.32**	0.45**	0.49**	0.56**	(0.92)

*N* = 642. S. D., Standard deviation; OrgGen, Organizational tenure; Type, Type of the team; SDF, Supervisor developmental feedback; PIden, Problem identification; TDF, Team developmental feedback; TR, Team reflexivity; Creat, Employee creativity. *p < 0.05, **p < 0.01. Two-tailed test.

### Measurement model tests

Before testing our hypotheses, we conducted CFAs to check if the studied variables captured distinctive constructs that they represent. We reported the CFA results of the hypothesized measurement model in Table 3. Our hypothesized five-factor model produced a good fit based on the widely-accepted cutoff criteria for fit indices (Hu and Bentler, 1999): *χ*^2^(289, *N* = 642) = 645.66, *p <* 0.001, *χ*^2^/df = 2.23, CFI = 0.96, TLI = 0.95, RMSEA = 0.05, SRMR = 0.04. We compared this baseline model with three alternative measurement models, as reported in Table 3. The chi-square difference test approach was used to compare the differences between our baseline model and the alternative measurement models. The best competing alternative model was the one in which we combined problem identification and employee creativity. The chi-square difference test suggested that our baseline measurement model produced significantly better fit than this best competing alternative model: Δ*χ*^2^ (4) = 630.30, *p <* 0.01. Therefore, our hypothesized measurement model captured distinctive constructs as expected. The alternative models showed a significantly worse fit than our baseline model.

**Table 3:** Comparison of measurement models based on confirmatory factor analyses.

Model	*χ* ^2^	df	Δ*χ*^2^	CFI	TLI	RMSEA	SRMR
Baseline model: five-factor	645.66	289		0.96	0.95	0.05	0.04
Alternative models							
1. Four-factor (PIden and creativity combined)	1275.96	293	630.30	0.88	0.87	0.09	0.08
2. Four-factor (SDF and TDF combined)	1640.56	293	994.90	0.85	0.83	0.10	0.10
3. Four-factor (TR and PIden combined)	1376.58	293	730.92	0.87	0.86	0.09	0.10
4. One-factor (All combined)	5493.14	299	4847.48	0.55	0.51	0.16	0.12

*N* = 642. PIden, Problem identification; SDF, Supervisor developmental feedback; TDF, Team developmental feedback; TR, Team reflexivity; *χ*^2^, chi-square; df, degree of freedom; CFI, comparative fit index; TLI, Tucker-Lewis index; RMSEA, Root mean square error of approximation; SRMR, Standardized root mean square residual.

### Hypotheses tests

#### 1-1-1 Model (hypotheses 1–3)

At the individual level, we intended to explore the relationship between SDF and employee creativity. We also assumed that problem identification would partially mediate such relationship because there might be a significant direct effect of SDF on creativity. As shown in Table 1, we used group-mean centering method to partition the within-team (level-1) and between-team (level-2) effects of predictors on creativity for 1–1-1 mediation model as recommended by Zhang et al. (2009). Our primary interest was to explore the level-1 mediation effects for this 1–1-1 model. At the individual level, we found significantly positive relationships between SDF and creativity (*γ =* 47, *p <* 0.01), supporting hypothesis 1. As shown in Table 4, we also found a significantly positive relationship between SDF and problem identification (*γ =* 0.25, *p <* 0.01). Hypothesis 2 was thus supported. Table 1 also indicate that there is a positive relationship between problem identification and creativity (*γ =* 44, *p <* 0.01), thus supporting hypothesis 3a. As recommended by Zhang et al. (2009), we estimated the indirect effect of SDF on creativity through problem identification by calculating γ_50(1)_ - γ_50(2)_, yielding an indirect effect size of *ρ* = 0.11 with a 95% CI of [0.05, 0.17] through the Monte Carlo method (Zhang et al., 2009). Thus, we also found support for hypothesis 3b.

**Table 4:** HLM results: the effects of developmental feedback on team reflexivity and problem identification.^a^

Variables	Team reflexivity	Problem identification	
	Model 1	Model 2	Model 3
Intercept	4.41**	4.43**	4.44**
Level-1 control variable			
Age, γ_10_	0.04**	0.02	0.01
Gender, γ_20_	0.05	0.03	0.03
OrgTen, γ_30_	0.00	−0.02	0.03
Edu, γ_40_	0.06*	−0.03	0.04
Level-1 predictor			
SDF, γ_50_		0.25**	0.25**
Level-2 control variable			
Type of team, γ_01_	0.04	0.09	0.04
Level-2 predictor			
TDF, γ_02_	0.33**		0.27**
Team Reflexivity, γ_03_			0.31**
Pseudo R^2b^	0.19	0.17	0.24

OrgTen, Organizational tenure; Edu, Educational level; SDF, Supervisor developmental feedback; TDF, Team developmental feedback. ^a^*N* = 642 at the individual level; *N* = 103 at the team level. ^b^Pseudo R^2^ was calculated based on the proportional reduction of Level 1 and Level 2 error variance due to predictors (Snijders and Bosker, 1999). *p < 0.05, **p < 0.01.

#### 2-1-1 Model (hypothesis 4)

We hypothesized that in addition to SDF at individual level, TDF (level-2) was also positively related to employee creativity and that such relationship was partially mediated by employees’ problem identification. Hypothesis 4a stated that team developmental feedback is positively related to creativity. As shown in Table 1, we found a significant relationship between TDF and employee creativity (*γ =* 16, *p <* 0.01), thus supporting hypothesis 4a.

In hypothesis 4b, we intended to explore the indirect effect of TDF on creativity through problem identification. First, as shown in Table 4, we found a significantly positive relationship between TDF and problem identification (*γ =* 27, *p <* 0.01). As recommended by Zhang et al. (2009), we group-mean centered the level-1 mediator, problem identification, and the level-2 variables in the full model with creativity as the dependent variable. This method stands in line with our theory because we intended to investigate how developmental feedback given by each team member (i.e., a team context of developmental feedback) might influence team members’ problem identification behavior and eventually produced creative outcomes. In other words, our primary interest was to explore the level-2 mediation effects in this 2–1-1 model, while the traditional grand-mean centering method in this case might produce confounded estimates of both level-1 and level-2 effects. We followed Zhang et al. (2009) approach and estimated the indirect effect of TDF on employee creativity through problem identification by calculating γ_02(model 6)_ - γ_02(model 7)_, yielding an indirect effect size of ρ = 0.12 with a 95% CI of [0.05, 0.19] through the Monte Carlo method (Zhang et al., 2009). Thus, we also found support for hypothesis 4b.

#### 2-2-1 Model (hypotheses 5–6)

We hypothesized that team reflexivity was a contextual factor (level-2) linking TDF and individual-level outcomes including both problem identification and employee creativity. In hypothesis 5a, we expected to find a positive relationship between TDF and team reflexivity. As shown in Table 4, TDF was positively related to team reflexivity (*γ =* 33, *p <* 0.01), thus providing support to hypothesis 5a. In the 2–2-1 models, we grand-mean centered level-1 variables and group-mean centered level-2 variables since centering method would not produce confounded estimates and thus would not bias the mediation effects (Zhang et al., 2009). As shown in Table 4, team reflexivity was found to be positively related to problem identification (*γ =* 31, *p <* 0.01). We used Monte Carlo method and obtained an indirect effect size of ρ = 0.10 with a 95% CI of [0.05, 0.16]. Thus, we also found support for hypothesis 5b.

As shown in Table 1, there was a significantly positive relationship between team reflexivity and employee creativity (*γ =* 24, *p <* 0.01), supporting hypothesis 6a. Hypothesis 6b stated that team reflexivity partially mediates the positive relationship between TDF and creativity. As recommended by Zhang et al. (2009), we estimated the indirect effect of TDF on creativity through team reflexivity by calculating γ_02(model 8)_ - γ_02(model 9)_, yielding an indirect effect size of ρ = 0.09 with a 95% CI of [0.04, 0.13] through the Monte Carlo method (Zhang et al., 2009). Thus, hypothesis 6b was also supported.

## Discussion

Drawing on the feedback and creativity literatures, this study develops and tests a multilevel model of developmental feedback and employee creativity. Using hierarchical linear modeling on data from 642 employees nested within 103 teams in a large Chinese telecommunication company, we show that both supervisor and team developmental feedback positively predict employee creativity. At the individual level, problem identification mediates the relationship between supervisor developmental feedback and creativity, whereas at the team level, team reflexivity serves as a key mediator linking developmental feedback to both problem identification and creativity.

### Theoretical implications

First, we directly theorize and test problem identification as a core mechanism linking developmental feedback to employee creativity, providing a more precise understanding of how and why feedback influences employee creativity. Although prior research has extensively examined the feedback–creativity relationship, the findings have been mixed (Chan et al., 2021; Ilies and Judge, 2005; Kim and Kim, 2020; Van Dijk and Kluger, 2011; Zhou, 1998; Zhou, 2003). Some studies suggest that the valence of feedback matters, with positive feedback promoting creativity (e.g., Chan et al., 2021) and negative feedback hindering it (e.g., Ilies and Judge, 2005; Van Dijk and Kluger, 2011; Zhou, 1998), while others highlight that even negative feedback can have constructive effects (e.g., Kim and Kim, 2020). We argue that these inconsistencies arise because previous work has largely overlooked the role of feedback in influencing the creative process itself (Amabile, 1983, 1996). By directly examining problem identification as a mediating mechanism, our study clarifies the underlying logic through which developmental feedback fosters employee creativity, thereby reconciling contradictory findings in the literature and advancing a process-oriented understanding of the feedback–creativity link.

Second, we advance prior work by adopting a multilevel framework to examine the effects of developmental feedback on creativity at both the employee and team levels. Previous studies have primarily focused on feedback from supervisors (e.g., Zhou, 2003; Zhang et al., 2019) or colleagues (e.g., Zhou and George, 2001) in isolation, thereby examining its impact on employee creativity from a single level of analysis. However, a key agreement in the creativity literature is that creativity does not “live in a vacuum” (Amabile, 1996; Shalley and Gilson, 2004; Shalley et al., 2004; Zhou and Shalley, 2003). Creativity is thus better understanded from an interactional system in which employees receive relevant resources and expert guidance from different-level factors (Madjar et al., 2002; Mumford et al., 2002; Tierney et al., 1999). By simultaneously investigating supervisor developmental feedback and team developmental feedback, our study captures the combined influence of individual- and team-level feedback on employee creativity, which contributes to theory building by extending feedback–creativity research beyond a single-level perspective.

Finally, we extend the feedback and creativity literature by demonstrating how team-level developmental feedback shapes employees’ problem identification and, in turn, their creativity, thereby introducing a cross-level mechanism that has received limited attention in prior research. Specifically, we incorporate team reflexivity as a key collective behavior that leverages the informational and motivational properties of the TDF to facilitate employee-level creativity. While previous studies on team reflexivity have primarily focused on team-level innovation (West, 1996, 2000), our research adds unique value by linking team reflexivity to employee creativity both directly and indirectly through employees’ engagement in problem identification within their own jobs. This multilevel perspective provides a richer understanding of how team-level processes influence employee creativity, offering a novel extension to the literature on feedback and creativity.

### Practical implications

In addition to the theoretical contributions, our multilevel findings offer actionable guidance for organizations seeking to foster employee creativity through developmental feedback. First, the research highlights the informational, motivational, and future-oriented nature of developmental feedback. This suggests that organizations should systematically establish growth-oriented feedback mechanisms. For example, through regular structured constructive dialogues, managers and employees can be guided to view feedback as a two-way process of exploring new possibilities, rather than a one-way evaluation. This approach can reduce the emotional burden often associated with feedback and enhance the generative and engaging nature of the interaction.

Second, the study indicates that team developmental feedback can stimulate creativity by promoting team reflexivity. Therefore, organizations can foster a culture of regular, constructive feedback within teams. Introducing mechanisms such as “team reflection sessions” can encourage members to exchange improvement suggestions in a trusting environment and jointly examine tasks and collaboration methods, thereby strengthening collective learning and an innovative atmosphere.

Furthermore, the research reveals the key mediating role of problem identification in the link between feedback and creativity. This implies that organizations should intentionally develop employees’ ability to analyze and reframe problems. For instance, through workshops or case-based training, employees can be helped to more systematically identify work challenges and explore innovative pathways during feedback interactions. Simultaneously, managers should maintain reasonable expectations during the feedback process, understanding the iterative and non-linear nature of creative development. Avoiding premature intervention or results-oriented anxiety can prevent undermining the supportive effect.

In summary, this study recommends that organizations focus on three areas—feedback mechanism design, team culture shaping, and cognitive skill development—to transform developmental feedback into a systematic practice that sustainably drives innovation for both individuals and teams.

## Limitation and future direction

Our study also suffered from several limitations. One important limitation of this study is its cross-sectional design, which does not allow for strong causal inferences. As we intended to explore the role of both individual (i.e., supervisor) and team developmental feedback in the production of individual creativity, our findings might be more causally supported if we would be able to capture the influence of developmental feedback on employee creativity over time. Yet, after partitioning level-1 and level-2 mediation effects, we were able to confirm three mediating mechanisms in our model. This alleviated our concern and suggested that the current study has captured the multilevel property of the relationship between developmental feedback and creativity. In line with the recent suggestions (Ployhart and Vandenberg, 2010), future research may consider adopting longitudinal, multi-wave, or experimental/quasi-experimental designs to more rigorously test our model.

Second, we did not collect and thus were not able to use objective ratings to reflect employee creativity. Prior research recommended the use of objective ratings on creativity to diversify empirical efforts in truly capturing the property of creativity at the workplace especially when the studied sample was nested in teams and performed tasks with shared characteristics or interdependent outcomes (Zhou and Shalley, 2003). Moreover, as we intended to clearly identify both the novelness and the usefulness of new ideas to justify the concept of creativity, it might be interesting to clearly investigate how developmental feedback might contribute to these two different elements which altogether define creativity. Thus, we advocate that future research consider collecting objective ratings from the company record on individual creativity to capture both the novelness and usefulness of new ideas in different task environments. Additionally, given that the sample of this study was drawn from a single organization, we encourage future research to replicate and extend the proposed model using multi-industry samples, as well as to investigate potential moderators that may influence the relationship between developmental feedback and creativity through the mechanism of problem identification.

Third, although we argued that TDF might initiate a repeated socialization process through which mutual-learning and improvement were accomplished through relational networking, in our study we did not reflect such socialization process in a developmental feedback context. Future research might incorporate personality variables and investigate how employees with different personalities might differ in interacting with both supervisors and other team members in the developmental feedback context. We believe that such differences in the interaction processes may influence individuals’ capability to utilize the rich developmental information they receive from multiple sources, thus contributing to their differences in generating new ideas that are closely aligned with the needs of the task environment and future challenges.

## Data Availability

The raw data supporting the conclusions of this article will be made available by the authors, without undue reservation.
